# The impact of the vaginal birth after cesarean as a new delivery mode on quality of life, sexual function, sleep quality and urinary incontinence during the postpartum period and comparison with the results of vaginal birth and cesarean delivery

**DOI:** 10.1002/ijgo.71150

**Published:** 2026-06-16

**Authors:** Berrak Bektaş, Can Ozan Ulusoy, Husamettin Uslu, Melda Kuyucu, Bulut Varli, Mehmet Efe Namlı, Pınar Özcan

**Affiliations:** ^1^ Department of Obstetrics and Gynecology Bezmialem Vakif University Medical Faculty Istanbul Turkey; ^2^ Perinatology Department Ministry of Health, Etlik City Hospital Ankara Turkey; ^3^ Department of Obstetrics & Gynecology Bruksel IVF & Endoscopic Laser Surgery Center Istanbul Turkey; ^4^ Department of Obstetrics and Gynecology Ankara University School of Medicine Ankara Turkey; ^5^ Department of Gynecology and Obstetrics Ministry of Health, Ankara Ataturk Sanatorium Research and Training Hospital Ankara Turkey; ^6^ Department of Obstetrics and Gynecology Istanbul Nişantaşı University School of Medicine Istanbul Turkey

**Keywords:** cesarean delivery, postpartum quality of life, sexual function, sleep quality, vaginal birth, vaginal birth after cesarean

## Abstract

**Objective:**

In the present study, we aimed to evaluate the impact of vaginal birth after cesarean (VBAC) on quality of life, sexual function, sleep quality, and urinary incontinence and compare the results with those of vaginal birth (VB) and cesarean delivery (CD) via validated questionnaires.

**Methods:**

A single‐center retrospective cross‐sectional observational study was conducted at the Bezmialem Vakıf University between 2017 and 2022. The participants were classified into three groups: VB (*n* = 49), CD (*n* = 54), and VBAC (*n* = 55). Postpartum maternal health outcomes were assessed via validated questionnaires: the Pittsburgh Sleep Quality Index (PSQI), Female Sexual Function Index (FSFI), Postpartum Quality of Life (PP‐QoL), and International Consultation on Incontinence Questionnaire‐Short Form (ICIQ‐SF). Statistical analyses were performed using SPSS version 29.0.

**Results:**

There were no significant differences among the groups in terms of total PSQI scores or ICIQ‐SF scores. However, the total FSFI and PP‐QoL scores were significantly higher in the VBAC group (*P <* 0.001). Among the FSFI subdomains, only desire and pain scores remained significantly different after Bonferroni correction.

**Conclusion:**

Our findings suggest that while sleep quality and urinary incontinence symptoms did not significantly differ among the delivery groups, PP‐QoL and FSFI scores were higher in the VBAC group than in the VB and CD groups.

## 1 INTRODUCTION

Childbirth is a significant event in a woman's life, both physically and psychologically, and it directly affects health, well‐being, and quality of life in the postpartum period. The mode of delivery is a key determinant of maternal physical recovery, sexual function, sleep quality, and overall well‐being. While vaginal birth (VB) is considered the most physiological process, cesarean delivery (CD) has become increasingly common worldwide because of medical indications or personal preferences. Vaginal birth after cesarean (VBAC) has emerged as an alternative option for women with a history of CD and is increasingly recommended. The American College of Obstetricians and Gynecologists (ACOG) stated that the majority of women with prior CD featuring a low‐transverse incision are suitable candidates for VBAC and should receive counseling regarding a trial of labor after CD.^1^

Quality of life is a complex concept that includes an individual's physical health, psychological well‐being, femininity, and sociocultural and educational conditions. Postpartum quality of life is an essential indicator of a mother's physical, emotional, and social well‐being. The impact of delivery modes, including VB, CD, or VBAC, on quality of life, sleep quality, sexual function, and urinary incontinence during the postpartum period, remains controversial. Given the physiological, psychological, and social effects of delivery mode, it is unsurprising that it negatively or positively affects the quality of life of women during the postpartum period. Compared with CD, VB is generally associated with a shorter recovery period and may contribute positively to overall quality of life.^2^ Limited data exist concerning the effect of the VBAC on postpartum quality of life.

Sexual function is affected by medical conditions and physical, psychological, and sociocultural factors. VB may negatively affect sexual function due to perineal trauma, episiotomy, and pressure during delivery, whereas CD is thought to preserve pelvic floor integrity and thus provide advantages. However, studies have not provided conclusive evidence to support this hypothesis.^3^ Furthermore, limited data exist regarding the effect of the VBAC on sexual function.

The impact of delivery mode on sleep quality is another critical factor influencing maternal well‐being in the postpartum period. Sleep disturbances have been associated with an increased risk of postpartum depression and anxiety.^4^, ^5^, ^6^, ^7^ VB is often associated with a faster recovery period, potentially leading to improved sleep quality. In contrast, CD may negatively affect sleep patterns because of postoperative pain and restricted mobility.^2^ However, the effect of the VBAC on sleep quality remains largely unexplored.

Insufficient data are available to assess the impact of the VBAC on postpartum quality of life, sexual function, sleep quality, and incontinence, but the relationship between delivery method and these factors constitutes a significant clinical concern. Limited evidence suggests that VBAC may be associated with maternal postpartum outcomes, but its relationship with postpartum quality of life, sexual function, sleep quality, and urinary incontinence remains insufficiently studied. Consequently, in the present study, we assessed the influence of the VBAC on quality of life, sexual function, sleep quality, and urinary incontinence and compared these outcomes with those of the VB and CD using validated questionnaires.

## 2 MATERIALS AND METHODS

This was a single‐center retrospective cross‐sectional observational study conducted at the Department of Obstetrics and Gynecology of Bezmialem Vakıf University Hospital between 2017 and 2022. Clinical and obstetric data were retrospectively collected between 2017 and 2022. The questionnaires were also routinely administered at the fourth postpartum month during this period as a part of the institutional clinical evaluation protocol for these patients. Written informed consent was obtained from all patients before the questionnaires. The study protocol was approved by the Ethical Committee of the Medical Faculty of Bezmialem University (E‐54022451, 21.02.2023). The questionnaire‐based assessment was subsequently conducted for the retrospective analysis of these previously collected clinical and questionnaire data after ethics committee approval. The participants were classified into three groups based on the mode of delivery: VB (*n* = 49), CD (underwent repeat CD because of previous history of a CD) (*n* = 54), and VBAC (*n* = 55).

The inclusion criteria were women aged 18–45 years who had undergone VB, CD, or VBAC at our hospital, had resumed sexual activity by the fourth postpartum month, and agreed to participate in the study. We excluded patients who had a known mental or neurologic disorder; had a sleep disorder that required treatment; were not willing to participate; worked night shifts; took medications that affect sleep patterns; or were diagnosed with pre‐eclampsia, eclampsia, chronic hypertension, intrauterine growth restriction (IUGR), or gestational diabetes mellitus (GDM) during pregnancy. Additionally, patients who had preterm delivery (<37 weeks of gestation), instrumental vaginal delivery (vacuum or forceps), or emergency cesarean delivery following labor were excluded. Women with failed VBAC resulting in intrapartum CD were also excluded. Because of the retrospective design, data on refusals and incomplete screening logs were not systematically available.

In this study, postpartum quality of life, sexual function, sleep quality, and urinary incontinence symptoms were evaluated via validated questionnaires. The Pittsburgh Sleep Quality Index (PSQI) was used to assess sleep quality,^8^ whereas the Female Sexual Function Index (FSFI) was used to evaluate sexual function.^9^ The International Consultation on Incontinence Questionnaire Short Form (ICIQ‐SF) was used to assess urinary incontinence symptoms. Postpartum quality of life (PP‐QoL) scores were also recorded.^10^ All questionnaires used in this study have been validated for the Turkish population by previous studies conducted by Ağargün et al. (PSQI), Öksüz and Malhan (FSFI), Tarcan et al. (ICIQ‐SF), and Altuntuğ et al. (PP‐QoL).^11^, ^12^, ^13^, ^14^ These questionnaires were administered once, at the fourth postpartum month, to participants who had resumed sexual activity.

The data collected included demographic characteristics of the patients (age, gravida, parity, abortion, smoking status, chronic disease history, and body mass index [BMI, calculated as weight in kilograms divided by the square of height in meters]), PSQI scores, FSFI scores, PP‐QoL scores, and ICIQ‐SF scores.

### 2.1 Statistical analysis

Statistical analyses were performed using IBM SPSS Statistics (IBM Corp., Armonk, New York). Descriptive statistics for continuous variables are expressed as mean ± standard deviation (SD) for normally distributed data, or as median (minimum–maximum) for non‐normally distributed data.

Continuous variables were assessed for normality using the Shapiro–Wilk test within each group. Variables meeting the normality assumption across all three groups were compared using one‐way analysis of variance (ANOVA) with Tukey HSD post hoc comparisons; non‐normally distributed variables were compared using Kruskal‐Wallis tests with Dunn‐Bonferroni post hoc pairwise comparisons. Categorical variables were analyzed using Pearson chi‐square tests or Fisher–Freeman–Halton exact tests when more than 20% of expected cell counts fell below 5. To account for baseline differences among groups in age, BMI, gravidity, and parity, four separate multiple linear regression models were constructed for the primary outcomes (FSFI total, PP‐QoL total, PSQI total, and ICIQ‐SF total). Delivery mode was dummy‐coded with VB as the reference group, and BMI category was dummy‐coded with ≤25 as the reference. Maternal age, gravidity, and parity were entered as continuous covariates. A Bonferroni correction was applied to account for multiple comparisons: *α* = 0.0125 for the four primary outcomes, *α* = 0.0083 for the six FSFI subdomains, and *α* = 0.0071 for the seven PSQI components. The general significance level was set at *P* less than 0.05 unless otherwise specified by Bonferroni correction. Power analysis was performed via G*Power 3.1 software to determine the minimum required sample size for comparing the means of three independent groups. Based on the data reported by Laganà et al. and assuming a significance level (*α*) of 0.05 and a desired statistical power (1–*β*) of 0.80, the minimum required sample size was calculated to be 54 participants in total (18 per group). This analysis ensured that the study was adequately powered to detect a statistically meaningful difference in FSFI scores between the groups.^15^

## 3 RESULTS

The baseline demographic and clinical characteristics of the groups are shown in Table 1. There was no significant difference in abortion, smoking status, or chronic disease history between the groups. A statistically significant difference was found between the groups in terms of age (*P* < 0.001), gravidity (*P =* 0.001), parity (*P* < 0.001), and BMI (*P* < 0.001). CD group was the youngest (27.9 ± 3.0 years), whereas VBAC group presented the highest gravidity (2.5 ± 1.2) (*P* = 0.001) and parity (2.2 ± 0.6) (*P* < 0.001). While most women in the VBAC and VB groups had a BMI between 26 and 30, a substantial proportion of women in CD group had a BMI between 31 and 35.

**TABLE 1:** Comparison of demographic and clinical characteristics among the vaginal, cesarean, and VBAC groups.

Parameters	Vaginal birth (*n* = 49)	Cesarean delivery (*n* = 54)	Vaginal birth after cesarean (*n* = 55)	*P* value
Maternal age (years)^a^ (mean ± SD)	30.4 ± 3.6	27.9 ± 3.0	29.3 ± 4.1	**<0.001** *
Gravidity (median; IQR)^b^	2.0 (1.0–2.0)	2.0 (1.0–2.0)	2.0 (2.0–2.0)	**0.001** *
Parity (median; IQR)^b^	2.0 (1.0–2.0)	1.5 (1.0–2.0)	2.0 (2.0–2.0)	**<0.001** *
Number of abortions (median; IQR)^b^	0.0 (0.0–0.0)	0.0 (0.0–0.8)	0.0 (0.0–0.0)	0.076
Smoking status^d^ *n* (%)	3 (6.1%)	4 (7.4%)	0 (0.0%)	0.135
Chronic disease^c^ *n* (%)	12 (24.5%)	13 (24.1%*)	9 (16.4%)	0.510
BMI at booking in kg/m^2^ groups^c^ *n* (%)				**<0.001** *
20–25	13 (26.5%)	9 (16.7%)	7 (12.7%)	
26–30	34 (69.4%)	23 (42.6%)	39 (70.9%)	
31–35	2 (4.1%)	22 (40.7%)	9 (16.4%)	

*Note*: BMI, calculated as weight in kilograms divided by the square of height in meters. Bold values indicate nominal statistical significance at *P* < 0.05.

Abbreviations: ANOVA, analysis of variance; BMI, body mass index; IQR, interquartile range; SD, standard deviation; VBAC, vaginal birth after cesarean.

^a^ One‐way ANOVA (F statistic).

^b^ Kruskal‐Wallis test (H statistic).

^c^ Pearson chi‐square test (*χ* ^2^ statistic).

^d^ Fisher–Freeman–Halton exact test (*χ* ^2^ statistic).

* Asterisks indicate statistical significance after Bonferroni correction where applicable.

In Table 2 the results for sleep quality, sexual function, postpartum quality of life, and urinary incontinence for all the groups are presented. There was a significant difference between the groups in terms of FSFI total scores and PP‐QoL scores. The total FSFI score was significantly greater in the VBAC group than in both VB and CD groups (*P* < 0.001), surpassing the Bonferroni‐corrected threshold. Post hoc Tukey HSD comparisons revealed that the VBAC group (27.46 ± 4.58) had significantly higher FSFI total scores than both the CD (24.06 ± 3.83, *P* < 0.001) and VB groups (25.23 ± 4.48; *P* = 0.025). No significant difference was observed between VB and CD (*P* = 0.355). Among the FSFI subdomains, a statistically significant difference was found between the groups in terms of desire (*P* < 0.001), satisfaction (*P* = 0.01), and pain (*P* < 0.001) scores. Significant group differences were identified for the desire subdomain (H [2] = 18.90, *P* < 0.001, *ε* ^2^ = 0.109), indicating a medium effect. Post hoc Dunn‐Bonferroni pairwise comparisons revealed that the VBAC group reported significantly higher desire scores than the CD group (*P* < 0.001), while no significant differences were found between VB and CD (*P* = 0.062) or between VB and VBAC (*P* = 0.169). The pain subdomain also showed significant group differences (H [2] = 23.47, *P* < 0.001, *ε* ^2^ = 0.139), representing a medium‐to‐large effect. Post hoc analyses indicated significant differences between VB and CD (*P* = 0.006) and between CD and VBAC (*P* < 0.001), with the CD group reporting lower pain subdomain scores compared to both VB and VBAC. No significant difference was found between VB and VBAC for pain (*P* = 0.375). The satisfaction subdomain approached but did not survive Bonferroni correction (H [2] = 9.31, *P* = 0.010; *α* = 0.0083).

**TABLE 2:** Comparisons of sleep quality, sexual function, postpartum quality of life, and urinary incontinence among the vaginal, cesarean, and VBAC groups.

Parameters	Vaginal birth (*n* = 49)	Cesarean delivery (*n* = 54)	Vaginal birth after cesarean (*n* = 55)	*P* value
PSQI score^2^	10.00 (7.00–11.00)	9.00 (7.00–10.75)	8.00 (5.50–11.00)	0.184
PSQI subdomains				
Subjective sleep quality^2^	2.00 (1.00–2.00)	2.00 (1.00–2.00)	1.00 (1.00–2.00)	**0.019** ^a^
Sleep latency^2^	1.00 (1.00–2.00)	2.00 (0.00–2.00)	1.00 (0.00–2.00)	0.214
Sleep duration^2^	2.00 (2.00–3.00)	2.00 (2.00–3.00)	2.00 (1.50–3.00)	0.509
Sleep efficiency^2^	1.00 (0.00–1.00)	1.00 (0.00–2.00)	1.00 (0.00–2.00)	0.294
Sleep disturbances^2^	2.00 (1.00–2.00)	2.00 (1.00–2.00)	1.00 (1.00–2.00)	0.099
Use of sleep medication^2^	0.00 (0.00–0.00)	0.00 (0.00–0.00)	0.00 (0.00–0.00)	0.382
Daytime dysfunction^2^	2.00 (1.00–2.00)	2.00 (1.00–2.00)*	1.00 (0.00–2.00)	**0.031** ^b,^ *
FSFI score^1^	25.23 ± 4.48	24.06 ± 3.83	27.46 ± 4.58	**<0.001** ^b,^ *
FSFI subdomains				
Desire^2^	3.60 (3.00–4.00)	2.90 (1.85–3.60)	4.20 (3.00–4.80)	**<0.001** ^c,^ *
Arousal^2^	3.97 (3.30–5.10)	4.20 (3.08–5.10)	4.50 (3.30–5.40)	0.305
Lubrication^2^	4.80 (4.20–5.40)	4.80 (4.20–5.40)	4.80 (4.50–5.70)	0.716
Orgasm^2^	4.40 (3.20–5.20)	4.30 (3.90–5.60)	4.40 (3.80–5.60)	0.160
Satisfaction^2^	4.80 (4.00–4.80)	4.80 (4.03–5.60)	5.20 (4.80–6.00)	**0.010** ^a^
Pain^2^	4.40 (3.60–5.60)	3.20 (2.00–4.35)	4.80 (4.40–6.00)	**<0.001** ^d,^ *
PP‐QoL score^1^	19.55 ± 5.20	21.18 ± 4.31	23.75 ± 3.98	**<0.001** ^b^ *
ICIQ‐SF score^2^	1.00 (1.00–4.00)	1.00 (1.00–4.00)	1.00 (1.00–5.00)	0.748
Frequency of urinary incontinence				0.279
Never	31 (63.3%)	27 (50%)	27 (49.1%)	
Once or several times a week	10 (20.4%)	18 (33.3%)	20 (36.4%)	
About once a day	3 (6.1%)	7 (13%)	3 (5.5%)	
Several times	5 (10.2%)	2 (3.7%)	5 (9.1%)	

*Note*: Values are presented as mean ± SD or median (IQR), as appropriate. One‐way ANOVA with Tukey HSD post hoc comparisons was used for normally distributed variables; Kruskal–Wallis tests with Dunn–Bonferroni post hoc comparisons were used for non‐normally distributed variables. The Fisher–Freeman–Halton exact test was used for categorical variables when expected cell counts were <5. Bonferroni‐corrected significance thresholds were *α* = 0.0125 for the four primary outcomes, *α* = 0.0083 for the six FSFI subdomains, and *α* = 0.0071 for the seven PSQI components. Bold values indicate nominal statistical significance at *P* < 0.05. Superscript letters indicate significant post hoc pairwise differences: a, Group 1 versus Group 3; b, Group 1 versus Group 3 and Group 2 versus Group 3; c, Group 2 versus Group 3; d, Group 1 versus Group 2 and Group 2 versus Group 3.

Abbreviations: ANOVA, analysis of variance; FSFI, Female Sexual Function Index; ICIQ‐SF, International Consultation on Incontinence Questionnaire‐Short Form; IQR, interquartile range; PP‐QoL, postpartum quality of life; PSQI, Pittsburgh Sleep Quality Index; SD, standard deviation; VBAC, vaginal birth after cesarean.

^1^ One‐way ANOVA with Tukey HSD post hoc comparisons was used for FSFI total and PP‐QoL. Kruskal–Wallis tests with Dunn–Bonferroni post hoc comparisons were used for PSQI, ICIQ‐SF, and subdomain analyses. Bonferroni‐corrected significance thresholds were *α* = 0.0125 for the four primary outcomes, *α* = 0.0083 for the six FSFI subdomains, and *α* = 0.0071 for the seven PSQI components.

^2^ Kruskal‐Wallis test. Post hoc: Dunn‐Bonferroni pairwise comparisons. 1–2 = VB versus CD; 1–3 = VB versus VBAC; 2–3 = CD versus VBAC. Bonferroni‐corrected *α* = 0.0083. *P < 0.0083.

* Asterisks indicate statistical significance after Bonferroni correction where applicable.

The PP‐QoL scores were significantly greater in the VBAC group (*P* < 0.001). PP‐QoL scores also differed significantly among the three groups (F [2, 155] = 11.59, *P* < 0.001, *η* ^2^ = 0.130), indicating a medium effect size. Post hoc analyses indicated that the VBAC group (M = 23.75, SD = 3.98) reported significantly higher PP‐QoL scores than both the CD (M = 21.18, SD = 4.31; *P* = 0.009) and VB groups (M = 19.55, SD = 5.20; *P* < 0.001).

There was no significant difference in total PSQI scores, ICIQ‐SF scores, or urinary incontinence between the groups. However, subjective sleep quality (*P* = 0.019) and daytime dysfunction (*P* = 0.031) were significantly greater in the vaginal birth group. Subjective sleep quality showed a trend toward significance (H [2] = 7.94, *P* = 0.019), as did daytime dysfunction (H [2] = 6.93, *P* = 0.031); however, neither survived the stringent Bonferroni correction. Other PSQI subdomains, including sleep latency, duration, efficiency, disturbances, and use of sleep medication, did not significantly differ between the groups. For urinary incontinence, the ICIQ‐SF scores did not differ significantly among the groups (*P* = 0.748). Similarly, the frequency of incontinence episodes (never, once or several times a week, once a day, or several times a day) did not significantly vary (*P* = 0.279).

Four separate multiple linear regression analysis were performed for each primary outcome (FSFI total, PP‐QoL, PSQI total, and ICIQ‐SF), incorporating the following covariates: maternal age (continuous), BMI category (dummy‐coded; ≤25 as reference), gravidity (continuous), parity (continuous), and delivery mode (dummy‐coded; VB as reference). After controlling for maternal age, BMI, gravidity, and parity, the multiple linear regression model predicting FSFI total scores was statistically significant (F [7, 150] = 3.48, *P* = 0.002, *R* ^2^ = 0.140, adjusted *R* ^2^ = 0.100), explaining approximately 14.0% of the variance in FSFI total scores. VBAC showed a significant positive association with FSFI total score (*β* = 2.175, 95% CI: 0.396, 3.954, *P* = 0.017), indicating that the VBAC group scored on average 2.17 points higher than the VB group after adjustment. The CD group did not differ significantly from VB (*β* = −1.693, *P* = 0.08). None of the covariates (age, BMI, gravida, parity) reached statistical significance in this model (all *Ps* >0.05). The regression model for PP‐QoL was also statistically significant (F [7, 150] = 4.98, *P* < 0.001, *R* ^2^ = 0.189, adjusted *R* ^2^ = 0.151), accounting for 18.9% of the variance. VBAC showed a significant positive association for PP‐QoL (*β* = 3.864, 95% CI: 2.029, 5.698, *P* < 0.001). Additionally, BMI 31–35 showed a significant positive association (*β* = 3.557, 95% CI: 1.215, 5.900, *P* = 0.003) for PP‐QoL, suggesting that women with higher BMI reported better postpartum quality of life after controlling for other variables. CD delivery did not differ significantly from VB (*P* = 0.43). The regression model for PSQI total scores did not reach overall significance (F [7, 150] = 1.75, *P* = 0.102, *R* ^2^ = 0.075), indicating that delivery mode and the covariates together did not significantly predict PSQI total scores. Nevertheless, maternal age emerged as a significant individual predictor (*β* = 0.197, 95% CI: 0.054, 0.339, *P* = 0.007), suggesting that each additional year of maternal age was associated with a 0.197‐point increase in PSQI score, independent of delivery mode. Neither CD (*β* = 0.485, *P* = 0.501) nor VBAC (*β* = −0.914, *P* = 0.177) differed significantly from VB. The ICIQ‐SF regression model was not statistically significant (F [7, 150] = 1.01, *P* = 0.427, *R* ^2^ = 0.045), indicating that neither delivery mode nor the covariates significantly predicted ICIQ‐SF. However, the regression models accounted for a modest proportion of the variance in the outcomes and residual confounding from unmeasured variables, particularly breastfeeding status may have affected the results.

## 4 DISCUSSION

In the present study, VBAC was associated with higher PP‐QoL and FSFI scores compared with the other delivery groups, whereas sleep quality and urinary incontinence measures did not significantly differ. However, we found that the desire scores were significantly higher in the VBAC group. Additionally, our results revealed that dyspareunia complaints were similar between the VB and VBAC groups, whereas pain scores were significantly lower in the CD group.

Several studies have evaluated postpartum sleep quality, urinary incontinence, sexual function, and quality of life after CD and VB by using assessment tools, including the PP‐QoL, FSFI, PSQI, and ICIQ‐SF. However, there is a lack of research specifically focusing on the results of these parameters after VBAC. The impairments in postpartum sexual function have been well documented in the literature, which supports our findings.^16^, ^17^, ^18^, ^19^, ^20^, ^21^, ^22^ A systematic review examining the effects of delivery mode on postpartum female sexual function reported that the mode of delivery itself does not directly impact postpartum sexual function. However, researchers have identified subjective birth experience and the degree of perineal trauma as potential moderating factors that could indirectly influence sexual function.^3^ Wiegel et al. reported that the optimal cutoff score for differentiating between women with and without sexual dysfunction for the FSFI scale is 26.55. Scores below this threshold indicate sexual dysfunction.^23^ In our study, the mean FSFI scores in the VB and CD groups were below this cutoff value, whereas the FSFI scores in the VBAC group were above the threshold, indicating a lower prevalence of sexual dysfunction in this group.

A study by Fobelets et al. demonstrated that women who preferred VB but underwent an elective repeat cesarean section had significantly lower health‐related quality of life (HR‐QoL) scores at three months postpartum.^24^ This finding may indicate that delivery preference and birth experience are relevant to PP‐QoL outcomes. In our study, the higher PP‐QoL scores observed in the VBAC group should be interpreted cautiously, as this association may have been influenced by unmeasured confounding factors rather than a direct psychological effect of VBAC.

A multicenter Life‐ON study, which included longitudinal assessments and polysomnographic data, demonstrated that sleep disturbances are highly prevalent in both the prenatal and postpartum periods.^25^ Moreover, a PSQI score above 5 has been found to identify 88.5% of individuals with sleep disorders. Ryali et al. assessed the relationship between sleep quality and VB or CD and reported that sleep disturbances were more common after CD.^2^ However, our study failed to find a relationship between the mode of delivery and sleep quality, while there is limited research on the VBAC in relation to sleep quality.

In our study, the mode of delivery, whether VB, CD, or VBAC, did not significantly affect the ICIQ‐SF scores or the frequency of urinary incontinence in the postpartum period. These findings contrast with those of previous studies. For example, Tähtinen et al. reported that compared with CD, VB nearly doubles the risk of long‐term stress urinary incontinence, with an absolute risk increase of approximately 8%, especially in younger women.^26^ Similarly, Varlas et al. reported that urinary incontinence, whether stress, urgency, or mixed type, is more frequently observed after VB because of its impact on pelvic floor support and innervation, with a reported 3% absolute increase in urgency incontinence compared with CD.^27^ However, it should be noted that neither of these studies specifically analyzed the VBAC as a separate group, and comparisons were limited to elective CD versus VB. In this context, our study provides additional value by including the VBAC as an independent group and evaluating its impact on urinary outcomes. Despite these expectations, no statistically significant differences were observed among the groups, suggesting that other factors may play a more dominant role in the development of postpartum urinary incontinence.

Our study is one of the pioneering investigations examining the effects of delivery mode on female sexual function, quality of life, and sleep disturbances, particularly from the perspective of the VBAC. The strengths of our study include subgroup analyses and single‐center setting, which ensured a homogeneous patient population with similar ethnic backgrounds. Additionally, the exclusion of emergency CD and instrumental vaginal deliveries minimized confounding factors. However, the first limitation of our study is that neonatal intensive care unit admissions were not evaluated, and paternal and neonatal factors that could have introduced confounding variables were not optimized. The second limitation is that the questionnaire‐based assessment was retrospectively analyzed with previously collected clinical and questionnaire data. Additional limitations include the lack of data on perineal trauma/episiotomy, birth weight, analgesia, breastfeeding status, known as a factor, related to prolactin‐mediated hypoestrogenism. These factors may have acted as residual confounders, particularly for postpartum sexual function. Moreover, the sample size calculation was performed based solely on FSFI outcome; therefore, the study was not formally powered to detect differences across all four primary endpoints including FSFI, PP‐QoL, PSQI and ICIQ‐SF. Despite these limitations, a major strength of our study is the simultaneous comparison of three delivery modes within the same clinical setting. Future research with larger sample sizes and optimized control for confounding variables is needed to further explore these findings.

## 5 CONCLUSION

The present study provides a comprehensive analysis of the effects of different delivery modes on postpartum sexual function, quality of life, sleep quality, and urinary incontinence with particular focus on VBAC. VBAC was associated with higher postpartum quality‐of‐life and sexual function scores, whereas sleep quality and urinary incontinence outcomes were comparable across groups. These findings should be interpreted cautiously in light of the retrospective cross‐sectional design and the potential residual confounding.

## AUTHOR CONTRIBUTIONS

P.Ö. and B.B. conceived and designed the study. P.Ö., B.B. and M.K. contributed to the methodology and study design. Data were collected by B.B and C.O.U., B.V., H.U. and M.E.N. contributed to investigation, data validation, and manuscript review. H.U. and P.Ö. drafted the initial version of the manuscript. P.Ö. supervised all stages of the study. All authors read and approved the final manuscript.

## FUNDING INFORMATION

The authors have nothing to report.

## CONFLICT OF INTEREST STATEMENT

The authors declare no conflict of interest.

## Data Availability

Research data are not shared.
